# Grant Funding for Immigrant Cardiovascular Health Research in the US and Canada

**DOI:** 10.1001/jamanetworkopen.2025.46110

**Published:** 2025-12-02

**Authors:** Wassim Bedrouni, Mahdi Bedrouni, Akl C. Fahed, Sonia S. Anand, Amal Bessissow, Rhian Touyz, Thao Huynh

**Affiliations:** 1 Research Institute of McGill University Health Centre, Division of Cardiology, Department of Medicine, McGill Health University Center, Montreal, Quebec, Canada; 2 Broad Institute of Massachusetts General Hospital and Harvard Medical School, Boston; 3 Department of Medicine Faculty of Health Sciences, McMaster University, Hamilton, Ontario, Canada; 4 Research Institute of McGill University Health Centre, Division of General Internal Medicine, Department of Medicine, McGill Health University Center, Montreal, Quebec, Canada; 5 Research Institute of McGill University Health Centre, Department of Medicine, Faculty of Medicine and Health Sciences, McGill University, Montreal, Quebec, Canada

## Abstract

This cross-sectional study examines the national grant funding for immigrant cardiovascular health research in the US and Canada from 2005 to 2023.

## Introduction

Both the US and Canada are experiencing substantial growth in immigrant populations.^1,2^ In addition to their differing cardiovascular (CV) risk profiles, immigrants face unique challenges in accessing health care services^1,2^ compared with nonimmigrants. It is of public health interest to evaluate the extent of national funding dedicated to cardiovascular health research of immigrants in the US and Canada.

## Methods

We searched the National Institute of Health (NIH) and the Canadian Institutes of Health Research (CIHR) web platforms for awarded grants that explicitly addressed cardiovascular health in immigrants from 2005 to 2023. We reported all funding in US dollars (USD). We defined an immigrant as a foreign national with legal permanent resident status. We normalized the funding per 1000 immigrants and computed the funding in proportions of the annual NIH and CIHR budgets and the gross domestic product (GDP). We examined the associations between the annual funding, immigrant populations, and GDP by linear regression. We compared funding using nonparametric analysis of variance testing in SPSS version 28 (IBM) and created the graphs in Excel version 16 (Microsoft). Statistical significance was set at *P* <.05, and tests were 2-sided. Data were analyzed from January 2024 to January 2025.

By institutional policy at McGill Health University, this research did not require ethical submission because there was no patient-related data compilation. This cross-sectional study followed the STROBE reporting guideline.

## Results

We identified 183 NIH and 189 CIHR research grants focusing on cardiovascular health in immigrants. As a percentage of the annual research agency budgets, the NIH spent a progressively increasing percentage from 0.03% in 2005 (total NIH budget: $28 billion; CV health in immigrants: 84 × 10^7^) to a peak of 0.04% in 2021 (total NIH budget: $41.5 billion; CV health in immigrants: 166 × 10^7^). Comparatively, the CIHR demonstrated an overall declining trend, decreasing to 0.02% of the total budget by 2021 (CIHR total budget: $1.32 billion, CV health in immigrants: 2.6 × 10^7^).

**Figure 1.**
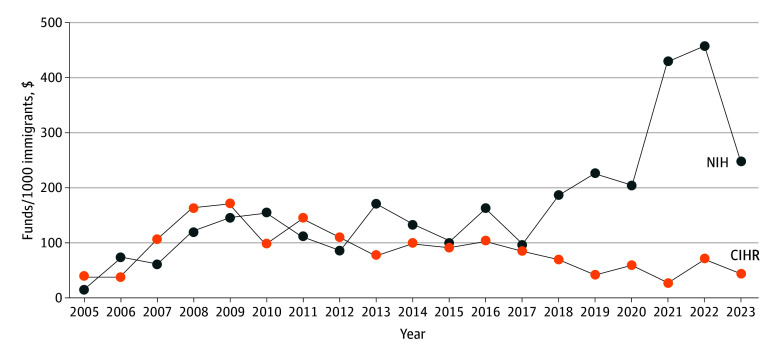
Annual Research Grant Funding for Cardiovascular Health CIHR indicates Canadian Institutes of Health Research; NIH, National Institutes of Health.

Since 2013, NIH spending adjusted for the number of immigrants consistently exceeded CIHR’s funding for the same purpose (Figure 1). The median (IQR) NIH annual spending (per 1000 immigrants) was $96.9 ($101.2-$198.0). In contrast, the median (IQR) CIHR yearly spending was $52.4 ($56.1-$108.5).

**Figure 2.**
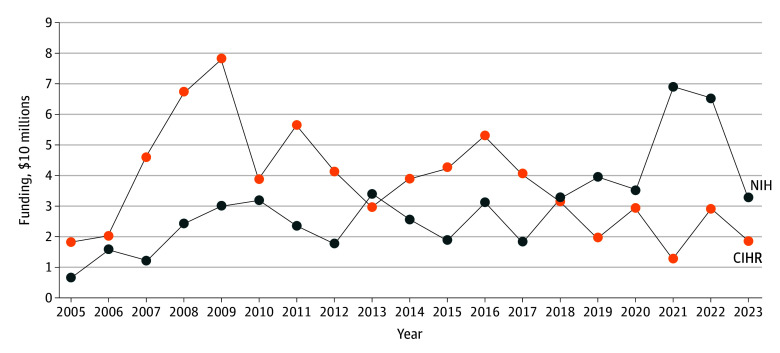
Annual Cardiovascular Health Research Grants as a Proportion of Gross Domestic Product CIHR indicates Canadian Institutes of Health Research; NIH, National Institutes of Health.

From 2005 to 2017, Canada dedicated a larger proportion of its national gross domestic product (GDP) toward cardiovascular health of immigrants than the US, after which the US outspent Canada as a percentage of GDP (Figure 2). The median (IQR) annual NIH expenditure was $3.88 ($2.49-$4.44) per $1 000 000 USD of GDP. The median (IQR) annual expenditure for CIHR was $3.00 ($1.85-$3.35) per $1 000 000 USD of GDP. We observed a strong linear correlation of the annual NIH funding for cardiovascular health in immigrants with the growth of the annual American GDP (*R* = 0.83; *P* < .001). In contrast, there was no association observed between CIHR spending and Canadian GDP.

## Discussion

During the last 2 decades, NIH consistently invested more than CIHR in cardiovascular health research in immigrants. This dominance in US national funding for cardiovascular health promotion among immigrants was observed in both the numbers of immigrants and GDP. Differences in research agency funding goals may contribute to these contrasting national trends. This may reflect a growing interest in health inequalities as reflected in the NIH strategic goals.^3^ In contrast, the CIHR did not explicitly express an interest in immigrants’ cardiovascular health.^4^

This study has limitations. We did not include projects focusing on refugees from minoritized backgrounds and individuals without legal immigration status. It was highly plausible that these communities have similar cardiovascular health inequalities as the immigrants. Finally, our results may no longer reflect the current situation of cardiovascular health research in the US due to the recent major budget cuts in research by the current federal government.^5^

In contrast to the sustained growth of US funding toward research for cardiovascular health of immigrants, we observed a reduction in Canadian research funding for the same purpose. Considering the continuous expansions in the number of immigrants, both the US and Canada should support more research focused on the cardiovascular health of these individuals.

## Notes

Supplement.
Data Sharing Statement
