# Supplementary material for: Grant Funding for Immigrant Cardiovascular Health Research in the US and Canada
Source: JAMA Netw Open. 2025 Dec 2;8(12):e2546110. doi: 10.1001/jamanetworkopen.2025.46110 (PMC12673409; doi:10.1001/jamanetworkopen.2025.46110)
Supplement: Supplement. — Data Sharing Statement [file jamanetwopen-e2546110-s001.pdf]

Data Sharing Statement

Data

Data available: Yes
Data types: Other (please specify)
Additional Information: cihr and nih list of grants approved for cardiovascular care in immigrants
How to access data: wassim.bedrouini@mail.mcgill.ca
When available: With publication

Supporting Documents

Document types: None

Additional Information

Who can access the data: researchers whose proposed use of the data has been approved
Types of analyses: for public health dissemination
Mechanisms of data availability: a signed data access agreement
Any additional restrictions: release of data should be agreed on by both the requester and the researcher
